# Respiratory muscle training improves aerobic capacity and respiratory muscle strength in youth wrestlers

**DOI:** 10.3389/fphys.2025.1492446

**Published:** 2025-03-27

**Authors:** Mehmet Ismail Tosun, Erkan Demirkan, Abdurrahim Kaplan, Yasemin Ari Yilmaz, Irem Eker Arici, Michael Favre, Veysi Aslan, Mehmet Kutlu

**Affiliations:** ^1^ Department of Physical Education and Sports, Faculty of Sport Sciences, Hitit University, Çorum, Türkiye; ^2^ Department of Movement and Training Sciences, Faculty of Sport Sciences, Hitit University, Çorum, Türkiye; ^3^ Department of Pulmonary Diseases, Faculty of Medicine, Hitit University, Çorum, Türkiye; ^4^ Intercollegiate Athletics, University of Michigan - Ann Arbor, Ann Arbor, MI, United States; ^5^ Department of Coaching Education, Graduate School of Health Sciences, Ege University, İzmir, Türkiye

**Keywords:** respiratory muscle training, wrestling, aerobic endurance, inspiratory muscle strength, peak inspiratory flow

## Abstract

**Background:**

Respiratory muscle training (RMT) has shown potential for enhancing athletic performance, but its effectiveness, in youth wrestlers, remains unclear. This study aimed to investigate the effects of RMT on respiratory muscle strength and aerobic endurance in youth wrestlers.

**Methods:**

A parallel-grouperal was conducted across 22 male youth wrestlers aged 14.8 ± 0.4 years. Participants were assigned to an experimental(E) group (n = 11), which received RMT in addition to their regular wrestling training, or a control(C) group (n = 11), which continued with standard wrestling training only. The RMT was performed three times a week using the POWERbreathe Classic Blue® device at 50% of maximal inspiratory pressure (MIP). Pre- and post-intervention measurements included MIP, peak inspiratory flow (PIF), inspiratory volume (IV), and aerobic endurance assessed by the Yo-Yo Endurance Level 1 test (YYT).

**Results:**

Significant improvements were observed in the E group, with MIP increasing by 9.57%, PIF by 14.77%, and IV by 10.46% (p < 0.05 for all). Aerobic endurance, as measured by VO_2_max and total running distance, also significantly improved by 4.93% and 8.22%, respectively (p < 0.05). The C group showed smaller yet significant gains in MIP, PIF, and VO_2_max but no significant change in IV.

**Conclusion:**

The addition of RMT to traditional wrestling training significantly enhances respiratory muscle strength and aerobic endurance in youth wrestlers. These results suggest that RMT may be an effective complementary training method to improve athletic performance in this population.

## 1 Introduction

With the development of the field of sports sciences, it has been observed that respiratory muscle training (RMT), one of the emerging types of training, has the potential to enhance athletic performance (Shen, 2023). Studies show that RMT is performed both in the acute phase and in chronic excess (Hartz et al., 2018; Lomax et al., 2011). However, when the results obtained are evaluated, it is argued that some study results support performance improvement, while others do not (Kilding et al., 2010; Sperlich et al., 2009). Therefore, it is important to conduct studies on different populations such as athletes or non-athletes, patients, etc. to eliminate this uncertainty. Especially in recent years, elite level athletic performance has reached its upper limits and records are difficult to break (Berthelot et al., 2015). Therefore, it feels warranted to support traditional methods with ergogenic training practices for performance improvement.

Wrestling is a unique combat sport that demands a combination of strength, power, endurance, agility, and technical skill. Unlike many other sports, it requires athletes to engage in repeated high-intensity efforts interspersed with short recovery periods, making both anaerobic and aerobic conditioning crucial (Chaabene et al., 2017). Its’ physiological demands are highly complex, as it integrates multiple energy systems to sustain performance under extreme physical exertion. Beyond physical fitness, wrestling also challenges an athlete’s mental resilience, reaction time, and strategic decision-making under fatigue (Kraemer et al., 2004). Given these requirements, optimizing conditioning strategies, including innovative training methods such as RMT, may offer a competitive advantage by enhancing endurance and recovery, ultimately leading to improved performance on the mat. Wrestling is a complex sport which regarding the duration of the competition, predominantly utilizes the anaerobic glycolytic energy system, requires the phosphagen system in explosive movements, and the aerobic energy system for the removal of increased lactic acid in the muscles and blood post-bout, as well as for rapid recovery (Demirkan et al., 2015). The effective use of each of the energy systems is one of the determining factors in the performance of youth wrestlers, especially in intense training and competition (Ratamess et al., 2016).

RMT enhances respiratory efficiency by strengthening the diaphragm and intercostal muscles, ultimately improving arterial oxygenation (Moreno et al., 2017). In young athletes, high Peak Inspiratory Flow (PIF) and Inspiratory Volume (IV) increase oxygen uptake capacity by strengthening respiratory muscles, which can directly contribute to performance (Bailey et al., 2010; Mickleborough et al., 2008). A high PIF value improves the oxygen delivery of oxygen to the muscles by allowing more oxygen to be transported to the alveoli. This meets the oxygen demand of the muscles, delaying fatigue and increasing endurance. A high IV value allows the respiratory muscles to take in larger volumes of air during inspiration, which increases arterial oxygenation and allows more oxygen to be delivered to the muscles (Lisboa et al., 1997). This process delays the accumulation of lactic acid in the muscles and prevents acidosis, allowing athletes to stay at the anaerobic threshold longer. Efficient respiratory muscle accelerate carbon dioxide excretion, maintaining pH balance and promoting rapid recovery (Merona et al., 2017). Improved oxygenation allows more oxygen to be transported to the muscles, which increases the efficiency of aerobic metabolism and causes a delay of lactic acid production above the anaerobic threshold (Messonnier et al., 2007). Strong respiratory muscle save energy by making the inspiratory process more economical for athletes. This energy saving can positively contribute to wrestlers’ performance by allowing them to direct more energy to their muscles during their competitions (Powers et al., 2007).

VO_2_max is one of the most important indicators of athletes’ aerobic capacity. A high VO_2_max allows athletes to supply more oxygen to their muscles during activity and utilize their energy efficiently (Bassett and Howley, 2000). In addition, VO_2_max value also affects the anaerobic threshold levels of athletes. Wrestlers with high VO_2_max and anaerobic threshold have more endurance in intermittent movements that require high intensity and explosive power during competition and can maintain their performance without feeling fatigue (Helgerud et al., 2007).

The mechanism of RMT can be explained by the fact that trained respiratory muscle efficiently assists to remove metabolic wastes more efficiently after intensive exercise, thus reducing lactate accumulation and accelerating recovery (Spengler et al., 1999). Strengthening the respiratory muscles and increasing VO_2_max increases the capacity of the circulatory system to carry more oxygen, increasing the amount of oxygen delivered to the muscles. This delays fatigue and increases endurance by delivering more oxygen to the muscles (Hinde et al., 2020; Held et al., 2020). Muscles that receive adequate oxygen supply can give wrestlers an advantage during competition and allow them to outperform their opponents. Strong respiratory muscle allow athletes to expend less energy during inspiration, which increases energy efficiency (Scano et al., 2006). This can improve the overall performance of wrestlers and contribute to a high level of performance, especially in the last rounds of competitions. By minimizing energy loss, wrestlers can maintain their endurance and maintain a physical edge in competition. Strong respiratory muscle allow carbon dioxide to be removed from the body faster, which can help blood pH to normalize quickly, and lactate to be transported more efficiently to the liver to participate in gluconeogenesis (Jones et al., 2002; Stickland et al., 2013). This may allow athletes to perform for longer periods of time, especially in high-intensity exercise (Sheel et al., 2001).

After an extensive examination of the literature, we found only one article investigating RMT on wrestling (Yoon and Jeon, 2011). This study specifically looked at the effects of such training on youth wrestlers. The aim of this study is to investigate the potential effects of RMT, in addition to routine wrestling practices, on respiratory muscle strength parameters such as maximal inspiratory pressure (MIP), PIF, IV, and aerobic endurance in youth wrestlers. The hypothesis of this study is that RMT, in addition to routine wrestling practices, will contribute to both respiratory parameters and the development of aerobic endurance.

## 2 Materials and methods

### 2.1 Participant

The required sample size was calculated with G* Power (Germany, version 3.1.9.6), with the level of significance set at α = 0.05, power (1 − β) = 0.95, and effect size ƒ = 0.25 (ANOVA with repeated measures, within-between interaction). According to the calculations, the total sample size was 16 participants. However, to account for possible dropouts and enhance the robustness of the findings, 26 participants were recruited. Since four have dropped out during the study, 22 were included in the final analysis (age 14.8 ± 0.4 years, height 168.6 ± 7 cm, body weight 67.6 ± 9.2 kg). The cards, numbered from 1 to 22 and placed one by one in capsules, were randomly selected by the participants after being mixed. Participants who chose numbers from 1 to 11 were assigned to the experimental (E) group (including number 11), and participants who chose numbers from 12 to 22 were assigned to the E group. Simple randomization type was used 11 youth wrestlers in the E group (age 14.7 ± 0.6 years, height 169.8 ± 4 cm, body weight 68.5 ± 11.9 kg, total body fat percentage 16.9 ± 4.06), and 11 youth wrestlers were included in the control (C)group (n = 11, age 14.9 ± 0.3 years, height 168.4 ± 7.9 cm, body weight 66.7.5 ± 5.9 kg, total body fat percentage 14.29 ± 2.5). A statistical analysis was conducted, and the results indicate that there were no significant differences between the groups in the pre-test measurements. All participants were freestyle wrestlers who trained under the same coach, followed the same training program, stayed at the same training camp, and adhered to the same nutrition plan within the same sports club. The inclusion criteria were: being between the ages of 14 and 16 (inclusive), valid medical certificate to compete in wrestling, lack of previous experience with RMT, at least 4 years of wrestling training. The exclusion criteria were: any acute or chronic medical condition and any ongoing medication intake. Before the study, approval was obtained from Hitit University Non-Invasive Research Ethics Committee (Protocol Number: 2024-80, Decision Number: 2024-04). In addition, the participants and their parents/guardians were given detailed information about the test-training procedures and possible risks. “Informed Parent/Guardian Consent Form” approved by Hitit University Non-Invasive Research Ethics Committee was obtained from the parents or guardians of the participants. The study commenced on March 4, 2024, and concluded on May 24, 2024. The Declaration of Helsinki was followed at all stages of the study.

### 2.2 Design and procedures

In this study, a parallel-group design was used to determine the effects of RMT on respiratory muscle strength and aerobic endurance in young wrestlers. Pre- and post-test measurements were completed in 1 day each. Two familiarization sessions were conducted 12 days before the pre-test measurements were taken to familiarize the participants with the tests administered. Both the Eand Cgroup participants underwent pre- and post-tests, administered in the following sequence. First, MIP was measured to assess inspiratory muscle strength. This was followed by the PIF test and the inspiratory volume test to evaluate inspiratory capacity. Subsequently, body fat percentage was assessed using bioelectrical impedance analysis (BIA). One hundred thirty minutes after the respiratory assessments aerobic capacity was evaluated using the Yo-Yo Endurance Level 1 test (YYT), and body fat percentage was reassessed with BIA. All tests were conducted in the specified order. All tests applied within the scope of pre-post test were applied by researchers specialized in the specified measurements. The same researchers who took part in the measurements were also blinded to the E/C group of the participants. All tests and RMT sessions were carried out under the supervisionof professional health specialist (MD) and sports science specialist (PhD) researchers. All tests were performed at least 48 h after the last training session, at the same time of day (10:00 - 11:00) and under the same environmental conditions (22°C, well ventilated, windless, indoor gym).

### 2.3 Measures

#### 2.3.1 Respiratory muscle strength (maximal inspiratory pressure)

Maximal inspiratory pressure is a valid method used to measure respiratory muscle strength. It reveals the capacity of inspiratory muscles to generate force during Muller maneuver (American Thoracic Society/European Respiratory Society, 2002; Volianitis et al., 2001). Maximum inspiratory pressure was determined with a portable device (Micro Medical-Carefusion Micro RPM, United Kingdom) (Dimitriadis et al., 2011; Stavrou et al., 2021). The measurement result can be monitored instantly on the device. Participants were asked to hold the measuring instrument with both hands and close their lips tightly around the flanged mouthpiece during the measurement. For the test, the participant was asked to exhale as deeply as possible (residual volume) followed by a maximum inspiration for more than one second (American Thoracic Society/European Respiratory Society, 2002). A nasal latch was applied to prevent air from escaping through the nose and the test was performed with the participants standing (Lomax et al., 2015). During the test, three measurements were taken, and the highest one was evaluated.

#### 2.3.2 PIF and inspiratory volume test

PIF assesses the ability of inspiratory muscles to contract rapidly and overcome a respiratory system-specific resistance (da Silva et al., 2023). Inspiratory volume is the volume and amount of air reaching the lungs on inspiration (Lee et al., 20016). A portable POWERbreathe® K5 device (POWERbreathe International Ltd., Warwickshire, England UK) was used for both assessments (Demirkan et al., 2025; Pupišová et al., 2014). The test was conducted while the participant was standing with their nose clipped shut. The participant was asked to exhale as much as possible with maximum expiration, immediately followed by maximum inspiration for more than 1 s. Both tests were performed at once in the single breath test mode of the device (Alvarenga et al., 2018).

#### 2.3.3 Body fat percentage (bioelectrical impedance analysis)

Body composition measurements of the participants were performed using a Tanita BC 418 segmental body composition analyzer (Tanita Corp., Tokyo, Japan). Measurements were performed in the morning, provided that participants had fasted for at least 12 h, and refrained from heavy physical activity for at least 48 h. During the measurement, participants were asked to remove all metal objects and step barefoot on the platform in light clothing (Demirkan et al., 2024; Kelly and Metcalfe, 2012).

#### 2.3.4 Measurement of aerobic capacity

The aerobic capacity of the participants was measured with the “Yo-yo Endurance Level 1” programme. Yo-yo Endurance Level 1 consists of 2 × 20 m shuttle runs at increasing speeds, with a 5-s active rest period between them (controlled by audio signals from a compact disc player). The test starts at a speed of 8 km/h and increases by 0.5 km/h approximately every minute. Participants continued the test until they were unable to maintain the required speed and distance. The point at which they were unable to maintain the required speed was recorded as the test result (Silva et al., 2011). Prior to the test, participants were instructed not to consume any performance-enhancing or stimulant supplements within the last 24 h, to maintain their standard dietary plan, and to avoid strenuous physical activities. The test result was converted to VO_2_Max with the calculator feature on the official website of the manufacturer that converts the yo-yo test to VO_2_Max (Yo-Yo Endurance Test Calculator - Convert YYET Score to VO_2_max, n.d.). The test was performed in an actively ventilated indoor gymnasium with a temperature of 22°C. The floor of the indoor sports hall used during the test was a wooden parquet floor (Yo-Yo Endurance Test Level 1, n.d.).

#### 2.3.5 Height (Stadiometer)

Participants’ height was measured using a SECA 213 (Made in Germany) with a measurement range of 20–205 cm and a measurement accuracy of 1 mm. Participants stepped barefoot on the disinfected area after each user and stood upright. The test result was recorded in cm.

### 2.4 Training protocol

#### 2.4.1 Respiratory muscle training

All participants in the study regularly trained wrestling 5 days a week. The exp. group underwent respiratory muscle training (RMT) using the POWERbreathe Classic Blue® (POWERbreathe International Ltd., UK) device for 12 weeks, three times per week (Monday, Wednesday, and Friday). RMT sessions were conducted 1 h before and 1 h after wrestling practice. RMT was performed as 30 repetitions with the POWERbreathe Classic Blue® device set at 50% of the participants’ MIP values (Griffiths and McConnell, 2007).

### 2.5 Statistical analysis

Three The normality of the distribution was evaluated with the Shapiro-Wilk test. Independent t-tests were conducted to compare participant characteristics between groups. The basic results are reported as mean and standard deviation. The effects for the interaction between time (before and after) and group (experimental and control) were analyzed using analysis of variance for repeated-measures. Effect sizes were calculated using partial eta squared (ηp^2^) and omega squared (ω^2^). A significance level of p < 0.05 was applied. All statistical analyses were performed using the JASP Team statistical package JASP (Amsterdam, Netherlands, version 0.17.2). The figures were prepared with GraphPad Prism (GraphPad Software, United States of America, version 10.1.2).

## 3 Results

**FIGURE 1:**
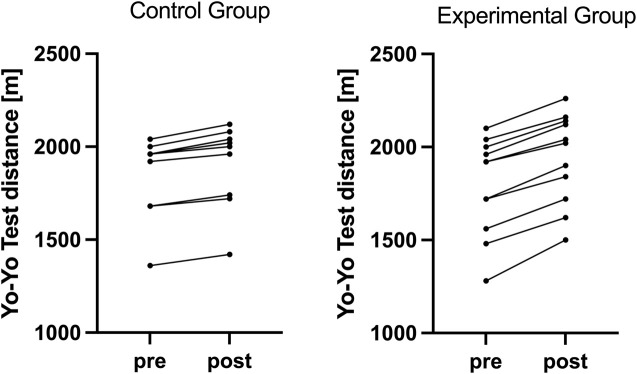
Individual changes in Maximum Inspiratory Pressure in both groups.

**FIGURE 2:**
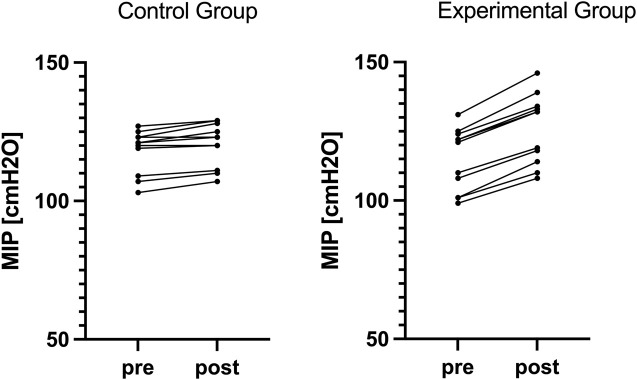
Individual changes in Yo-Yo Test distance in both groups.

Significant differences in changes across multiple respiratory and performance variables were observed between E and C groups, as presented in Table 1. There was a statistically significant difference regarding changes in MIP between the training groups (F = 110.450, p < 0.05, ηp2 = 0.847, ω2 = 0.044), with larger increases in MIP in E compared to C group (Figure 1). Moreover, significant difference concerning changes in PIF was observed between the training groups (F = 59.259, p < 0.05, ηp2 = 0.748, ω2 = 0.089), with larger increases in PIF in the group undertaking additional RMT. There was a statistically significant difference for changes in IV between the training groups (F = 19.425, p < 0.05, ηp2 = 0.493, ω2 = 0.119), with larger increases in IV in E compared to C group. Finally, all the performance improvements were significantly larger in E compared to C group (F = 56.461, p < 0.001, ηp2 = 0.739, ω2 = 0.009 for YYT distance, and F = 39.460, p < 0.05, ηp2 = 0.664, ω2 = 0.008 for VO_2_max) (Figure 2).

**TABLE 1:** Respiratory parameters, VO_2_max, and yo-yo test distance values of the experimental and control groups.

Value	Experimental group			Control group			p value	Effect size	
	Pre-test	%	Post-test	Pre-test	%	Post-test		pη^2^	ω^2^
MIP (cmH_2_O)	114.9 ± 11.4	9.57	125.9 ± 12.6	118.0 ± 7.9	2.07	120.5 ± 7.8	<0.05	0.847	0.44
PIF (L/s)	6.09 ± 0.66	14,77	6.99 ± 0.80	6.35 ± 0.34	2,67	6.52 ± 0.44	<0.05	0.748	0.089
Volume (liters)	2.77 ± 0.19	10.46	3.06 ± 0.21	2.87 ± 0.09	1.74	2.92 ± 0.11	<0.05	0.493	0.119
YYT Distance (meter)	1790.91 ± 261.59	8.22	1938.18 ± 244.04	1861.82 ± 205.02	3.32	1923.64 ± 210.86	<0.05	0.739	0.009
VO_2_max (mL/kg/min)	46.77 ± 4.12	4.93	49.08 ± 3.69	47.9 ± 3.18	2.1	48.91 ± 3.17	<0.05	0.664	0.008

*p* < 0.005. MIP: maximal inspiratory pressure; PIF: peak inspiratory flow is the maximal flow rate; IV: Inspiratory volume (liters); VO_2_max: Maximal ability of muscles, lungs, etc. to absorb, deliver and utilize oxygen during the exercise; YYT, Distance: The total distance completed during the yo-yo test; ml: milliliter; kg: kilogram; min: minute.

## 4 Discussion

To the best of our knowledge, this is the first study to investigate the effects of traditional wrestling training plus RMT on respiratory variablesand aerobic endurance in youth wrestlers. Therefore, our first aim was to investigate the effects on inspiratory muscle strength, and the second aim was to determine the effects on aerobic endurance. The main findings of the present study supported our hypotheses. Our primary findings indicated a significant improvement in all respiratory and performance indices in E compared to C group (Table 1).

In the previous studies performing RMT in athletes, Romer et al. (2002) reported that a 6-week RMT program provided higher improvement in MIP and PIF than the placebo group in trained cyclists. Bailey et al. (2010) performed inspiratory muscle training during the 4 weeks on sixteen healthy subjects, concluding that RMT increased MIP according to the baseline values and decreased inspiratory muscle fatigue. Notter et al. (2023) performed 2 different inspiratory muscle training methods in healthy young men demonstrating that both methods enhanced respiratory muscle parameters, but without supporting whole-body exercise performance during running and cycling. Similarly, previous studies have reported that in elite and well-trained athletes, RMT generally produces significant improvements in respiratory parameters, while its effects on other performance indicators remain inconclusive (Kowalski et al., 2024; Espinosa-Ramírez et al., 2023). In contrast to these results, Mickleborough et al. (2008) in a study on the effects of RMT in elite swimmers reported that the results appeared to have no evident difference between undergoing swimming training alone and swimming training plus RMT. Athletic performance improvement decreases as athletes reach their limit points due to their performance being limited by physiological and genetic conditions (Berthelot et al., 2015; Gulbin et al., 2013). The possible explanation related to this condition is that elite athletes could be on the upper limits of performance due to the study conducted on elite competitive swimmers (Mickleborough et al., 2008). Such studies were not limited to athletic populations exclusively.

Importantly, RMT induces additional training load. Kowalski et al. (2023) conducted a randomized controlled trial to examine the physiological stress imposed by RMT in well-trained triathletes, comparing voluntary isocapnic hyperpnea and inspiratory pressure threshold loading. Their findings demonstrated that RMT introduces an extra stress, with significant variations based on the training method and sex of the participants. Specifically, voluntary isocapnic hyperpnea was associated with greater perceived training load, whereas inspiratory pressure threshold loading elicited disruptions in acid-base balance, elevated blood lactate concentrations, and adverse symptoms such as headaches and dizziness. These results underscore the differential physiological effects of RMT methodologies in elite athletes, highlighting the necessity for careful implementation and individualized adaptation in high-performance training programs. Ferraro et al. (2019) explored the effects of RMT using two different protocols (30 breaths twice daily at ∼50% of MIP, and 60- breaths once daily at ∼15% MIP) in healthy older adults. The authors concluded that both groups had significant improvements in MIP, but the groups using ∼50%, had a greater improvement as compared to the groups that did ∼15% (45.9%, and 18.0% respectively). Bosnak-Guclu et al. (2011) reported that RMT enhanced functional capacity, and respiratory and peripheral muscle strength in patients with heart failure. Alvarenga et al. (2018) stated that the combination of inspiratory muscle training with Pilates exercises resulted in improved lung function and physical conditioning of elderly patients. In a similar study, Elmorsi et al. (2016) found significant improvements in inspiratory muscle strength in Chronic obstructive pulmonary disease patients using RMT plus exercise compared to exercise-only patients with. Souza et al. (2014) reported that a protocol of 8 weeks of RMT in elderly women improves MIP and PIF values, together with diaphragm thickness, and mobility. Our study results are in agreement with previous study findings, with exception to the study conducted by Mickleborough et al. (2008). Our results suggest that this training method may be performed with a combination of traditional training to enhance inspiratory muscle strength in athletes. In another study that was conducted on racehorses by Katz et al. (2020), the authors reported that RMT could be performed to maintain and/or increase aspects of resting inspiratory muscle strength in racehorses during the detraining period that is not in active exercise training.

In terms of endurance performance, both training groups in our study exhibited improvements in VO_2_max, though the E group RMT group demonstrated significantly greater gains in VO_2_max and covered distance compared to the greater gains in VO_2_max and covered distance compared to the C group. The reason could be explained by the addition of RMT. These findings were supported by several studies, to include one conducted by Lomax et al. (2011), that confirmed the combination of inspiratory muscle warmup and inspiratory muscle training increased the distance covered (14.9 + 4.5%) during an intermittent running test to exhaustion. During exercise, the mechanical load on the respiratory muscle directly influences both the ventilation-perfusion relationship and the distribution of oxygen to skeletal muscles. Enhanced efficiency of the respiratory muscle in meeting increased ventilatory demands delays respiratory fatigue. Improvements in respiratory muscle endurance allow ventilation to be maintained at a lower energy cost, thereby increasing the proportion of oxygen available for skeletal muscle function (McConnell, 2012; Esposito and Ferretti, 2010). The postponement of respiratory muscle fatigue suppresses metabolic reflex activity, contributing to a more optimal distribution of blood flow to peripheral muscles (Sheel et al., 2001; St Croix et al., 2000). Consequently, vasoconstrictive responses triggered by sympathetic activation during exercise are reduced, preserving perfusion to working muscles. More efficient oxygen utilization, facilitated by enhanced mitochondrial activity within muscle fibers, supports aerobic energy production and prolongs endurance capacity (Hansen et al., 2000). Improvements in gas exchange dynamics are achieved through reduced respiratory frequency and increased tidal volume. Enhanced alveolar ventilation helps maintain pulmonary diffusion capacity at an optimal level, thereby preserving arterial oxygen saturation (Toneloto et al., 2008; Yang and Yang, 2002). In parallel, the elevation of ventilatory thresholds mitigates respiratory strain during submaximal exercise, optimizing the balance between energy production and expenditure (Lomax et al., 2015).

These physiological adaptations provide a significant advantage in endurance performance. The reduction in perceived exertion during exercise allows individuals to sustain higher intensities for longer durations while also increasing the total distance covered. In this context, training the respiratory muscle directly enhances both ventilatory and metabolic efficiency, ultimately improving overall exercise performance. However, the authors reported that the effect of inspiratory muscle training alone was higher (12 + 4.9%) compared to that of an independent inspiratory muscle warm-up. In another study, Elmorsi et al. (2016) reported RMT plus exercise enhanced the covered distance in the 6-min walking test compared to the exercise-only group. Bailey et al. (2010) stated that 4 weeks of RMT led to enhanced V02 dynamics and exercise tolerance during severe and maximal-intensity exercise. Romer et al. (2002) confirmed that RMT decreased the perceptual response to maximal incremental exercise and improved performance in both the simulated 20 and 40 km time trials in trained male cyclists. Tong et al. (2016) stated that a total of 10 weeks of RMT in an interval training program provided significantly higher improvements in 1-h running time-trial performance and running economy in recreational runners. Based on these findings, it appears that our study results are in agreement with the previous findings except for a study conducted by Notter et al. (2023). The reason for the opposite of findings (Mickleborough et al., 2008; Notter et al., 2023) may be due to the differences in study procedure and/or the groups. Other than these particular papers, the majority of the studies (Alvarenga et al., 2018; Bailey et al., 2010; Bosnak-Guclu et al., 2011; Elmorsi et al., 2016; Ferraro et al., 2019; Katz et al., 2020; Lomax et al., 2011; Romer et al., 2002; Souza et al., 2014; Tong et al., 2016) are in agreement with our study results. Besides, in many previous studies (Cheng et al., 2013; Barnes and Ludge, 2021; Cheng et al., 2020; Gobatto et al., 2022; Lomax et al., 2011; Tanriverdi et al., 2021; Marostegan et al., 2022; Özdal et al., 2016; Tong and Fu, 2006; Wilson et al., 2014; Volianitis et al., 2001) demonstrated not only RMT, but also respiratory muscle warm-up exercise provided an increase in acute performance and/or fast recovery in athletes.

## 5 Conclusion

The present study results indicate that the application of a 12-week RMT plus traditional wrestling enhances MIP, PIF, IV values, and also aerobic endurance simultaneously. Therefore, we suggest that RMT may be a potential training method to strengthen respiratory muscle and assist in improving aerobic endurance in youth wrestlers. These findings further support the notion that RMT may serve as an effective training modality to enhance respiratory muscle function and contribute to improved aerobic endurance in young wrestlers. Based on these results, it is recommended that RMT 3 times a week together with traditional training may be set up for the training programs in junior wrestlers. Further research should clarify whether RMT should be applied simultaneously to elite and non-elite athletes to determine the effect of athletic level on development.

## 6 Limitation

Some limitations of our study should be acknowledged. First, the study was conducted on a relatively specific population of developing male athletes, which limits the generalizability of the findings to other populations, such as female athletes, patients, or sedentary individuals. Notably, athletes generally exhibit superior ventilatory efficiency (Kasiak et al., 2024), and research on RMT in female athletes has often reported greater positive adaptations compared to males (Kowalski et al., 2024). Additionally, VO_2_max was not directly measured but rather estimated from the YYT. While this method provides a practical alternative, conducting gold-standard cardiopulmonary exercise tests could have provided deeper insights into specific physiological adaptations, such as exercise thresholds or the effects of RMT on ventilation parameters. Furthermore, although participants were randomly assigned to groups, a formal randomization procedure was not implemented. However, baseline assessments revealed no significant differences in any measured variable between groups (p > 0.05). Finally, the RMT protocol did not incorporate progressive overload. Given that the intervention lasted 12 weeks, it is likely that MIP increased over time. Prior studies have demonstrated MIP improvements in athletic populations within as little as 4 weeks of RMT (Johnson, 2007; Chang et al., 2021). Consequently, in the later weeks of the intervention, the applied training load may have become insufficient, failing to align with the initial training assumptions.

## Data Availability

The raw data supporting the conclusions of this article will be made available by the authors, without undue reservation.
